# Trends and Impact of *Clostridioides difficile* Infection on Thirty-Day Readmissions and Outcomes Following Coronary Artery Bypass Grafting: A Seven-Year National Analysis

**DOI:** 10.14740/gr2099

**Published:** 2026-02-04

**Authors:** Abhin Sapkota, Ninda Sherpa, Maria Grba, Fernando Sigala, Ernesto Luna, Gedion Yilma Amdetsion, Hemant R. Mutneja, Vikram Kotwal

**Affiliations:** a Department of Medicine, John H. Stroger Jr. Hospital of Cook County, Chicago, IL 60612, USA; b Rush Medical College, Rush University Medical Center, Chicago, IL 60612, USA; c Division of Gastroenterology, John H. Stroger Jr. Hospital of Cook County, Chicago, IL 60612, USA

**Keywords:** Clostridioides, Coronary artery bypass, Mortality, Patient readmission, Sepsis

## Abstract

**Background:**

*Clostridioides difficile* infection (CDI) is a common hospital-acquired infection, particularly seen among patients who undergo coronary artery bypass grafting (CABG). This study aimed to assess the trends and impact of CDI on 30-day readmissions and outcomes in CABG patients.

**Methods:**

This retrospective analysis used the Nationwide Readmissions Database from 2016 to 2022. Patients undergoing CABG were identified using International Classification of Diseases, 10th Revision (ICD-10) procedure codes. Patients with CDI were identified using ICD-10 diagnosis codes. Multiple logistic regression was performed to adjust for confounding factors. Trend analysis was done.

**Results:**

A total of 1,279,605 adult patients undergoing CABG were included, of whom 5,567 (0.44%) had concurrent CDI. Patients with CDI were older (mean age 68.6 vs. 66.3 years, P < 0.001) and had higher medical comorbidity (Charlson Comorbidity Index ≥ 3: 68.4% vs. 45.35%, P < 0.001). They also had significantly higher rates of 30-day readmissions (12.63% vs. 7.35%, adjusted odds ratio (aOR), 1.42), in-hospital mortality (11.64% vs. 2.58%, aOR, 2.72), and complications including cardiac arrest (7.56% vs. 2.04%, aOR, 2.55), cardiogenic shock (26.89% vs. 9.50%, aOR, 2.33), sepsis/septic shock (26.05% vs. 2.51%, aOR, 8.28), all with P < 0.001. Over the study period, there was a decline in CABG admissions. CDI incidence among CABG patients also declined with a parallel decline in 30-day readmissions.

**Conclusions:**

Patients who underwent CABG with concurrent CDI experienced greater readmission rates, mortality, periprocedural adverse events, and resource utilization. Further studies need to be done for possible interventions to reduce these outcomes.

## Introduction

Coronary artery bypass grafting (CABG) is among the most frequently performed cardiac procedures for patients with advanced coronary artery disease, offering improved survival and enhanced quality of life [1, 2]. Advances in surgical techniques, perioperative care, and postoperative management have led to improved outcomes; however, CABG continues to be associated with significant perioperative morbidity, with infectious complications representing a substantial proportion of postoperative adverse events [3-6]. Among hospital-acquired infections, *Clostridioides difficile* infection (CDI) remains one of the most commonly reported and clinically consequential [7]. Several risk factors predispose patients undergoing CABG to acquire CDI, including perioperative exposure to broad-spectrum antibiotics, advanced age, multiple medical comorbidities, and extended hospital stays [8, 9].

Despite a nationwide decline in CDI incidence, including hospital-acquired cases, its burden persists in vulnerable populations—particularly among patients undergoing major cardiac procedures such as CABG [10]. In this population, CDI has been previously linked to increased mortality and elevated healthcare utilization [11, 12]. Prior studies have shown that in patients undergoing cardiac surgery, CDI is associated with increased hospital lengths of stay, higher rates of sepsis, renal dysfunction, and respiratory complications [13, 14]. However, much of the existing evidence is derived from single-center or retrospective studies, often limited by small sample sizes and lack of diversity. While large administrative datasets such as the National Inpatient Sample have helped characterize some in-hospital outcomes, they are limited by the absence of readmission data. Consequently, there remains a significant gap in understanding post-discharge outcomes, particularly readmission rates in this high-risk population.

To address this gap, we utilized the Nationwide Readmissions Database (NRD), a nationally representative, all-payer administrative dataset that contains detailed information on hospitalizations and readmissions for millions of patients across the United States. We compared demographic and clinical characteristics, in-hospital complications, and 30-day readmission outcomes between CABG patients with and without CDI. Additionally, we assessed healthcare resource utilization, including hospital length of stay (LOS) and total hospitalization charges.

## Materials and Methods

### Data source

A retrospective cohort analysis was conducted using the Agency for Healthcare Research and Quality’s Healthcare Cost and Utilization Project (HCUP) NRD for 2016 to 2022. The NRD is the largest publicly available, all-payer inpatient care readmission database in the United States. It is designed as a stratified probability sample to be representative of nationwide inpatient hospitalizations. To obtain this data, hospitals are initially stratified according to ownership/control, urban/rural location, number of beds, teaching status, and geographic region. A 20% probability sample of all hospitals within each stratum is then collected. Each discharge is then weighted (weight = total number of discharges from all acute care hospitals in the United States divided by the number of discharges included in the 20% sample) to make the NRD nationally representative. The dataset includes comprehensive patient- and hospital-level information, including demographics, admission and discharge diagnoses, procedures performed, LOS, readmission status, and total hospitalization costs. The NRD captures up to 40 diagnostic codes and 25 procedure codes per admission, coded using the International Classification of Diseases, Tenth Revision, Clinical Modification (ICD-10-CM). Because the NRD includes only deidentified data, this study was considered exempt from Institutional Review Board oversight under federal regulation 45 CFR 46.102(e). In accordance with institutional policy, the study was reviewed and determined to be not human subject’s research. All analyses were conducted in compliance with the HCUP Data Use Agreement. This study was conducted in compliance with the ethical standards of the responsible institution on human subjects as well as with the Helsinki Declaration.

### Study population

Patients who underwent CABG during hospitalization were identified using the ICD10-CM procedure codes (02100, 02110, 02120, 02130) and included in the study. Patients were excluded if they were younger than 18 years old. Because the NRD captures admission purely on a calendar year basis from January 1 through December 31 without a link to the previous or following year, index hospitalization discharges occurring in December were also excluded. Among those included in the study, patients who had a diagnosis of CDI were identified using ICD10-CM (A047). The research project is exempt from Institutional Review Board approval because it is a retrospective review of already collected deidentified data.

### Study outcomes

The primary outcome was 30-day all-cause hospital readmission. Each patient included in the NRD is assigned a unique database identification number. This number can be used to identify all admissions within the state for each patient during each calendar year from 2016 to 2022. A readmission was defined as any non-traumatic admission for any principal diagnosis within 30 days of the index admission. If patients had multiple readmissions within 30 days of discharge, only the first readmission was counted.

The secondary outcomes were 1) in-hospital mortality rate for index admissions; 2) ventilator use for more than 24 h (ICD10-CM: 5A09457, 5A09557); 3) cardiac arrest (ICD10-CM: I46); 4) surgical site infection (ICD10-CM: T8141XA, T8142XA, T8143XA, T8149XA); 5) post-procedure sepsis/septic shock (ICD10-CM: A41, R652, T8144XA, T8112XA); 6) acute kidney injury (AKI, ICD10-CM: N17); 7) vasopressor use (ICD10-CM: 3E043XZ); 8) blood transfusion (ICD10-CM: 30233N1); 9) resource use associated with readmission: length of hospital stay and mean total hospitalizations charges in US dollars.

### Definition of variables

NRD variables were used to identify demographic factors including age (in years), sex, insurance type (Medicare, Medicaid, private insurance, and self-pay), median household income for patient’s zip code ($1–$55,999, $56,000–$70,999, $71,000–$93,999, and $94,000 or more), hospital region (Northeast, Midwest, South, West), hospital bed size (small: 1 to 49 beds, medium: 50 to 99 beds, and large: more than 100 beds), and hospital location and teaching status (nonmetropolitan, metropolitan teaching, and metropolitan non-teaching). Age was stratified into age groups (18 to 65 years and more than 65 years).

The comorbidity burden was assessed using Deyo’s modification of the Charlson Comorbidity Index (CCI). CCI is an empirical prediction of 1-year mortality, based on the number and severity of comorbid diseases by using a weighted index [15]. The CCI was categorized into four groups (CCI category 0, CCI category 1, CCI category 2, and CCI category 3 or more). Other individual comorbid diagnoses were also identified and were used in the logistic regression analysis to reduce confounding effect, including: 1) hypertension (ICD10-CM: I10); 2) diabetes mellitus (ICD10-CM:E10, E11); 3) obesity (ICD10-CM: E66); 4) chronic kidney disease (ICD10-CM: N18); 5) hyperlipidemia (ICD10-CM: E78); 6) heart failure (ICD10-CM: I50); 7) chronic obstructive pulmonary disease (ICD10-CM: J41, J42, J43, J44); 8) tobacco use disorder (ICD10-CM: F17, Z720).

### Statistical analysis

Analysis was done using HCUP Project survey data analysis packages, incorporating NRD-specific variables, including stratum, hospital identifiers, and discharge weights to account for clustering and large survey-weighted data analysis to obtain statistical and variance calculations. These calculations are independent of individual hospital discharge characteristics. Weighted analyses were used to produce national estimates, and multivariable logistic regression models were employed to assess adjusted odds ratios (aOR) for primary and secondary outcomes among CABG patients with and without CDI. These approaches allowed us to account for the complex survey design of the NRD and to estimate nationally representative, adjusted associations between CDI and postoperative outcomes.

The analysis was adjusted for age, sex, medical comorbidities in the form of CCI and individual comorbidities as mentioned above, median household income quartile, insurance status, hospital region, hospital bed size, and hospital location/teaching status. A P value of less than 0.05 was considered statistically significant. All analyses were performed using STATA, version 18 (StataCorp, TX).

## Results

### Patient demographics and characteristics

A total of 1,279,605 adult patients undergoing CABG during index hospital admission were included in the analysis. Among these patients, 5,567 (0.44%) had concomitant secondary diagnosis of CDI. Baseline demographic and hospital characteristics of the study cohort are shown in Table 1. Patients with CDI were slightly older than those without CDI (mean age 68.6 vs. 66.3, P < 0.001). There was also a higher percentage of patients with CDI older than 65 (68.87% vs. 59.84%, P < 0.001). Males had a higher percentage of CABG admissions with and without CDI, but females comprised a greater proportion of the CDI group compared to the non-CDI group (35.19% vs. 24.23%, P < 0.001). Patients with CDI also had higher medical complexity with a higher percentage having a CCI of 3 or more compared to those without CDI (68.4% vs. 45.35%, P < 0.001) (Table 1).

**Table 1:** Demographic Characteristics of the Study Cohort

Variables	CABG admissions without CDI (n = 1,274,038)	CABG admissions with CDI (n = 5,567)	P value
Mean age in years (standard error)	66.32 (66.26–66.38)	68.58 (68.16–69.00)	< 0.001
Age groups			
18 to 65 years	511,606 (40.16%)	1,733 (31.13%)	< 0.001
More than 65 years	762,431 (59.84%)	3,834 (68.87)	< 0.001
Gender			
Male	965,361 (75.77%)	3,608 (64.81%)	< 0.001
Female	308,676 (24.23%)	1,959 (35.19%)	< 0.001
Charlson Comorbidity Index			
0	146,344 (11.49%)	234 (4.20%)	< 0.001
1	271,851 (21.34%)	632 (11.35%)	< 0.001
2	278,019 (21.82%)	892 (16.02%)	< 0.001
3 or more	577,822 (45.35%)	3,808 (68.40%)	< 0.001
Hospital bed size			
Small	123,128 (9.66%)	502 (9.02%)	0.350
Medium	306,171 (24.03%)	1,231 (22.11%)	0.055
Large	844,738 (66.30%)	3,834 (68.87%)	0.022
Hospital location and teaching status			
Non-metropolitan	31,631 (2.48%)	142 (2.55%)	0.858
Metropolitan non-teaching	185,475 (14.56%)	780 (14.01%)	0.466
Metropolitan teaching	1,056,931 (82.96%)	4,646 (83.46%)	0.555
Insurance			
Medicare	735,456 (59.91%)	3,912 (72.80%)	< 0.001
Medicaid	90,780 (7.40%)	381 (7.09%)	0.564
Private insurance	372,517 (30.34%)	995 (18.52%)	< 0.001
Self-pay	28,805 (2.35%)	85 (1.59%)	0.042
Income quartile			
1st quartile	342,190 (16.76%)	1,541 (17.23%)	0.375
2nd quartile	363,756 (17.83%)	1,547 (17.29%)	0.425
3rd quartile	637,149 (31.21%)	2,854 (31.92%)	0.472
4th quartile	698,182 (34.20%)	3,001(33.56%)	0.707

CDI: *Clostridioides difficile* infection; CABG: coronary artery bypass grafting.

In terms of hospital characteristics, most CABG admissions occurred in large, metropolitan teaching hospitals among both groups. However, a higher proportion of CDI patients were admitted to large hospitals compared to patients without CDI (68.9% vs. 66.3%, P = 0.022). There were no significant differences noted among hospital teaching status, rurality, or income level. There was a similar proportion of people within each income quartile that underwent CABG with or without associated CDI. However, notable differences were observed among primary insurance types. Medicare coverage was more common among CDI patients compared to patients without CDI (70.27% vs. 57.73%, P < 0.001). In contrast, private insurance was more common among patients without CDI (29.24% vs. 17.87%, P < 0.001) (Table 1).

### Postoperative outcomes and complications

**Figure 1:**
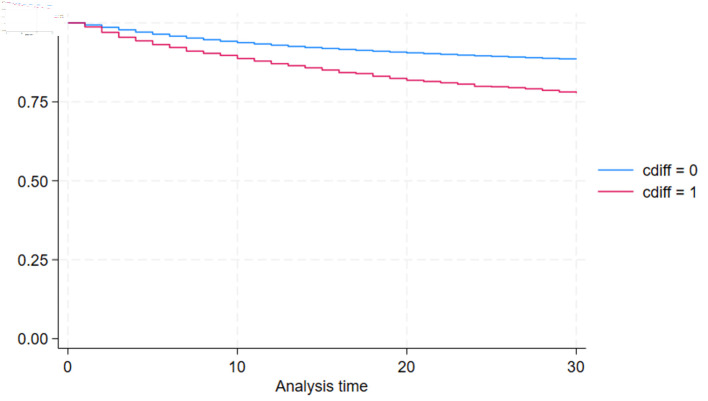
Kaplan-Meier curve depicting the 30-day readmission proportion of patients with and without CDI (blue line: CABG admission without CDI, red line: CABG admission with CDI). CDI: *Clostridioides difficile* infection; CABG: coronary artery bypass grafting.

Postoperative complications and clinical outcomes during the index hospitalization are summarized in Table 2. Patients undergoing CABG with a secondary diagnosis of CDI experienced significantly higher rates of complications across nearly all measured outcomes. Thirty-day readmissions were significantly higher in CDI patients compared to patients without CDI (12.63% vs. 7.35%, P < 0.001). Figure 1 shows Kaplan-Meier curve depicting the 30-day readmission proportion of patients with and without CDI indicating higher proportion of 30-day readmissions in CDI group. In-hospital mortality was also higher in the CDI group compared to the non-CDI group (11.64% vs. 2.58%, P < 0.001) (Table 2).

**Table 2:** Complications and Outcomes During Indexed Hospital Admission for CABG

Outcomes	Total hospitalizations with CABG, age > 18 (n = 1,279,605)			
	Without *C. diff* (n = 1,274,038)	With *C. diff* (n = 5,567)	Adjusted OR (95% CI)	P value
30-day readmissions	93,653 (7.35%)	703 (12.63%)	1.42 (1.26–1.60)	< 0.001
Mortality	32,917 (2.58%)	648 (11.64%)	2.72 (2.37–3.12)	< 0.001
Ventilator use > 24 h	9,659 (0.76%)	76 (1.37%)	1.46 (1.34–1.64)	0.017
Cardiac arrest	26,032 (2.04%)	421 (7.56%)	2.55 (2.16–3.00)	< 0.001
Cardiogenic shock	120,981 (9.50%)	1,497 (26.89%)	2.33 (2.10–2.58)	< 0.001
Surgical site infection	2,994 (0.24%)	57 (1.02%)	2.41 (1.58–3.67)	< 0.001
Post-procedure sepsis/septic shock	31,996 (2.51%)	1,450 (26.05%)	8.28 (7.36–9.32)	< 0.001
Acute kidney injury	278,837 (21.89%)	3,039 (54.59%)	2.88 (2.59–3.20)	< 0.001
Vasopressor use	72,798 (5.71%)	532 (9.56%)	1.46 (1.26–1.68)	< 0.001
Blood transfusion	157,674 (12.38%)	1,218 (21.88%)	1.41 (1.27–1.56)	< 0.001
	Mean differences (95% CI)			
Mean length of stay in days	10.34 (10.27–10.41)	27.10 (26.18–28.02)	–	< 0.001
Mean total hospitalization charge ($)	249,633.7 (245,046.1–254,221.3)	548,493 (523,685.8–573,300.2)	–	< 0.001

CABG: coronary artery bypass grafting; *C. diff*: *Clostridioides difficile* infection; CI: confidence interval; OR: odds ratio.

The percentage of organ dysfunction and critical illness was also substantially higher in patients with CDI. Patients with CDI experienced a higher need for prolonged mechanical ventilation beyond 24 h compared to patients without CDI (1.37% vs. 0.76%, aOR = 1.46, P < 0.017). Furthermore, patients with CDI were more than twice as likely to experience cardiac arrest (7.56% vs. 2.04%, aOR = 2.55, P < 0.001) and cardiogenic shock (26.89% vs. 9.50%, P < 0.001) compared to patients without CDI (Table 2).

CDI was also strongly associated with surgical site infections and post-procedure sepsis. Although relatively rare, surgical site infections occurred in a higher percentage of CDI patients compared to non-CDI patients (1.02% vs. 0.24%, P < 0.001). Postoperative sepsis or septic shock also occurred more in patients with CDI than those without CDI (26.05% vs. 2.51%, P < 0.001). Interestingly, AKI was highly prevalent in both groups with it occurring in over half of the patients in the CDI group (54.59% vs. 21.89%, P < 0.001). Finally, vasopressor use was significantly higher in the CDI group (9.56% vs. 5.71%, P < 0.001), as well as the need for blood transfusion during admission (21.88% vs. 12.38%, P < 0.001) (Table 2).

The most common primary diagnoses at 30-day readmission among patients with and without CDI are presented in Table 3. Among CABG index admissions without CDI, the most common primary diagnosis at readmission was hypertensive heart disease with heart failure (8.21%), followed by combined hypertensive heart and chronic kidney disease (6.21%). In contrast, patients with CDI had markedly different readmission patterns, with sepsis of unspecified organism accounting for nearly one-third of readmissions (29.72%), followed closely by CDI-related enterocolitis (23.89% for unspecified recurrent and 19.91% for non-recurrent cases) (Table 3).

**Table 3:** Top Primary Diagnoses at Readmission in CDI and Non-CDI

Serial number	ICD-10 code	Name	Total (% total)
	CABG without CDI (n = 93,653)		
1	I110	Hypertensive heart disease with heart failure	7,690 (8.21%)
2	I130	Hypertensive heart disease and CKD with heart failure and stage 1 through stage 4 CKD, or unspecified CKD	5,816 (6.21%)
3	J90	Pleural effusion, not elsewhere classified	4,805 (5.13%)
4	A419	Sepsis, unspecified organism	4,203 (4.48%)
5	I2699	Other pulmonary embolism without acute *cor pulmonale*	3,016 (3.22%)

Serial number	ICD-10 code	Name	Total (% total)
	CABG with CDI (n = 703)		
1	A419	Sepsis, unspecified organism	209 (29.72%)
2	A0472	Enterocolitis due to *Clostridioides difficile*, not specified as recurrent	168 (23.89%)
3	A047	Enterocolitis due to *Clostridioides difficile*	140 (19.91%)
4	I130	Hypertensive heart and CKD with heart failure and stage 1 through stage 4 CKD, or unspecified CKD	40 (5.68%)
5	A414	Sepsis due to anaerobes	37 (5.26%)

The top five primary diagnoses for readmission among a total of 94,356 readmissions are listed. CDI: *Clostridioides difficile* infection; CABG: coronary artery bypass grafting; ICD-10: International Classification of Diseases, 10th Revision; CKD: chronic kidney disease.

### Healthcare resource utilization

Patients undergoing CABG with CDI require more healthcare resources than those without CDI. The average LOS for patients with CDI was 27.1 days compared to 10.3 days in patients without CDI (P < 0.001). Not surprisingly, the mean total hospitalization charges were more than double in patients with CDI compared to patients without CDI ($548,493 vs. $249,634, P < 0.001) (Table 2).

### Trends in CABG admissions and CDI incidence

From 2016 to 2022, there was an overall annual decline in patients admitted for CABG. This decrease in CABG admissions became starker in 2020, suggesting a possible relation with the coronavirus disease 2019 (COVID-19) pandemic and rescheduling of non-urgent surgeries, though this variation was not statistically significant on trend analysis. Following 2020, there was an increase in admissions for CABG that plateaued but remained lower overall than the years preceding 2020. The prevalence of CDI notably declined from 2016 to 2019 before plateauing after 2020, and this decrease over time was noted to be statistically significant (P < 0.001). There was also a decline in 30-day readmission rates from 2016 to 2022, which was statistically significant (P < 0.001), and paralleled the decline in CDI incidence (Table 4).

**Table 4:** Number of CABG Admissions and Incidence of CDI (2016–2022)

Year	Total CABG admissions (P = 0.670)	Total CDI among CABG admission (% total) (P < 0.001)	30-day readmissions among all index admissions (% total) (P < 0.001)
2016	194,278	1,347 (0.69%)	16,732 (8.61%)
2017	194,059	1,016 (0.52%)	14,966 (7.71%)
2018	187,899	883 (0.47%)	14,129 (7.52%)
2019	190,966	673 (0.35%)	13,871 (7.26%)
2020	161,450	506 (0.31%)	11,232 (6.96%)
2021	175,536	591 (0.34%)	11,824 (6.74%)
2022	175,417	551 (0.31%)	11,602 (6.61%)

CDI: *Clostridioides difficile* infection; CABG: coronary artery bypass grafting.

## Discussion

This large, nationally representative analysis of 1,279,605 hospitalizations for CABG from the NRD revealed a CDI incidence of 0.44%. The presence of CDI in patients undergoing CABG was significantly associated with higher rates of readmissions, worse in-hospital outcomes, greater resource utilization, and higher mortality. Our findings are consistent with and expand upon prior literature, while adding new information about readmission data in this population. Similar increases in mortality and hospital LOS have been associated with CDI in cardiac surgery patients [13, 16]. Notably, we also observed a strong relationship between CDI and post-procedure sepsis/septic shock (aOR, 8.28), supporting previous work suggesting that CDI may serve as a marker for systemic inflammatory complications and organ dysfunction [16, 17]. Via a larger sample size and comprehensive analysis, our study provides a more robust and nuanced understanding of the association between CDI and CABG outcomes, in addition to more updated estimates of these risks that further emphasize the importance of preventing CDI in patients undergoing CABG. Importantly, however, the associations observed in this study should not be interpreted as evidence of causality. Given the retrospective and observational nature of the NRD, CDI may represent a marker of greater postoperative illness severity rather than a direct driver of adverse outcomes. Patients with complicated postoperative courses, prolonged hospitalizations, and greater exposure to broad-spectrum antibiotics may be inherently more susceptible to CDI. Accordingly, our findings demonstrate strong associations rather than causal relationships.

### Incidence and trends

The observed CDI incidence of 0.44% in our CABG cohort falls in line with previously reported rates following cardiac surgery, ranging from 0.5% to 0.93%, with smaller cohort studies reporting rates as high as 1.2% [8, 13, 18]. Notably, we observed a consistent decline in the incidence of CDI over the study period. This trend may reflect stricter infection control practices and antimicrobial stewardship efforts, such as the 2016 US Food and Drug Administration (FDA) black box warning to fluoroquinolones. This is further supported by the findings of a multicenter study conducted in 2017 that showed a statistically significant decrease in the rate of hospital-onset CDI following fluoroquinolone restriction [19]. Another potential consideration for the decreasing incidence of CDI over time could be due to heightened infection control measures and institutional hypervigilance during and following the COVID-19 pandemic [20].

### Risk factors

Patients with CDI were more likely to be older, female, and carry a greater comorbidity burden, as reflected by the higher CCI scores. These demographic trends mirror earlier reports and highlight known risk factors for CDI, including advanced age and immunocompromised states [13, 21]. Among hospital-based risk factors, there was a significantly higher incidence of CDI in CABG patients in larger hospitals, compared to smaller hospitals though there was no significant difference in CDI status based on rurality or hospital teaching status.

### Outcomes

One of the strengths of our study is its comprehensive assessment of systemic complications and in-hospital outcomes associated with CDI. We found significantly increased odds of multiple serious events such as sepsis/septic shock, cardiac arrest, cardiogenic shock, and AKI. These findings are consistent with prior research where a nearly fourfold increase in composite adverse events after cardiac surgery was reported in patients with CDI [13], while another study found increased rates of septicemia, prolonged mechanical ventilation (> 24 h), renal failure, and reoperation for bleeding in patients with CDI [16]. Our study builds on this evidence by identifying additional complications that have not been previously described.

This comprehensive outcome analysis offers clinicians better insights into the potential role of CDI within a broader cascade of postoperative complications. This observation prompts further inquiry into whether CDI serves as an independent contributor to postoperative complications or simply as a marker of heightened morbidity in the post-CABG setting. For instance, the higher rate of cardiac complications in the CDI group could reflect increased susceptibility to CDI and a higher incidence of CDI due to prolonged and complicated postoperative courses, or CDI may function as a marker of postoperative illness severity rather than a direct cause of these complications.

It is important to consider the underlying pathophysiology of CDI and how it may drive effects beyond the gastrointestinal tract. The inflammatory cascade in CDI is primarily driven by toxins TcdA and TcdB, which disrupt cytoskeletal structure and tight junctions in intestinal epithelial cells, leading to cell death, inflammation, and diarrhea. It is known to induce a robust systemic inflammatory response, characterized by elevated pro-inflammatory cytokines (interleukin (IL)-6, IL-8, IL-1β, tumor necrosis factor (TNF)-α) and increased inflammatory mediators (myeloperoxidase, prostaglandin E2) [22]. The systemic dissemination of *Clostridioides difficile* toxins has been shown to correlate with disease severity and fatal outcomes, as these toxins induce pro-inflammatory cytokine production that contributes to both local and systemic manifestations of the infection [23].

These biologic mechanisms suggest how CDI may influence organ systems outside the gastrointestinal tract and contribute to complications not typically associated with gastrointestinal infections. Case reports have linked CDI to Takotsubo cardiomyopathy, suggesting potential cardiotoxic and extraintestinal effects of the *Clostridioides difficile* toxin [24], while animal studies have demonstrated toxin-induced disruptions of cardiac contractility and rhythmicity [25]. We further hypothesize that certain outcomes such as shock, AKI, and vasopressor use may be secondary to the relative volume depletion resulting from CDI. Additionally, the increased incidence of sepsis and surgical site infections in the CDI group could reflect immune dysregulation triggered by CDI. Together, these findings may help explain some of our study’s results and further highlight the need for additional preventative strategies, and early recognition and treatment of CDI. While inflammatory cascades and toxin-mediated systemic effects of CDI are biologically plausible, these mechanistic explanations should be interpreted as hypothesis-generating rather than causal. Moreover, extrapolating these mechanisms to a cardiac surgical cohort must be done cautiously, as the present analysis lacks temporal resolution and cannot directly evaluate whether these processes precede or follow postoperative complications.

The principal outcome of our study was the rate of readmission among CABG patients who had concurrent diagnosis of CDI. We found that patients undergoing CABG who also had concomitant diagnosis of CDI during their hospitalization had 42% greater odds of readmission compared to those without CDI. There was a previous study that found a threefold increase in 30-day readmission rates among CDI patients after cardiac surgery [8]. While readmissions after CABG are often related to common complications like postoperative infections, arrhythmias, and heart failure [26], our study demonstrates how CDI may help identify a subgroup of CABG patients at particularly high risk for readmission. The increased readmission rates noted in the CDI group in our study likely reflect the higher rates of post-surgical complications like sepsis/septic shock, cardiac arrest, cardiogenic shock and AKI that were seen in CDI group, in addition to the baseline risk of readmission in this vulnerable patient population.

Among index CABG admissions, those who had concomitant CDI were more frequently readmitted for infectious causes compared to those without CDI. In the CDI group, four of the five most common reasons for readmission were infection-related, including various types of sepsis and enterocolitis. In contrast, readmissions in the non-CDI cohort were primarily cardiopulmonary in etiology, likely reflecting post-surgical complications, though sepsis was still the fourth most common cause for readmission in this group. These findings represent starkly different readmission profiles between the two groups, suggesting a greater infection burden post-discharge in patients with CDI. However, the temporal relationship between CDI and readmission-prompting complications cannot be established using the NRD. CDI may occur late during the index hospitalization or reflect underlying clinical deterioration that predisposes patients to both infection and subsequent readmission, rather than directly mediating readmission risk. Accordingly, the observed association should be interpreted as identifying heightened vulnerability rather than implying causality.

Furthermore, we found that there were nearly three times higher odds of mortality in CDI patients who underwent CABG than those without CDI. Several studies have reported similar odds (aOR, 2.24 [13]; aOR, 2.0 [11]), while one analysis in 2007 did not find a significant difference in 30-day mortality between the two groups [17]. Long-term follow-up studies have demonstrated that the mortality disadvantage persists beyond the immediate postoperative period, with lower 3-year survival rates among those with CDI than those without [16].

Lastly, we found that CDI was associated with significantly longer hospital stays (mean LOS 27.1 vs. 10.3 days) and over twofold higher hospitalization costs ($548,493 vs. $249,634). These findings emphasize the economic burden CDI imposes on healthcare systems, echoing previously described results, with similarly inflated resource use among cardiac surgery patients with CDI [27]. Prolonged hospitalization, greater procedural complexity, and higher severity of illness may increase susceptibility to CDI through extended antibiotic exposure and intensive care unit (ICU) stays, rather than CDI being the primary driver of longer LOS and higher costs. Accordingly, CDI may function as a surrogate marker of prolonged and complicated postoperative courses rather than a direct cause of increased resource utilization.

### Prevention

Our results clearly demonstrate the poorer outcomes related to CDI in patients undergoing CABG. Prior studies have highlighted the role of prolonged or broad-spectrum perioperative antibiotics in increasing CDI risk [8, 21]. Given that cardiac surgery often necessitates pre- and postoperative antibiotic use, our findings support the need for enhanced antimicrobial stewardship, and additional preventative measures in this setting. Historically, preventive strategies have focused on improved hand hygiene, contact isolation, environmental decontamination, and antibiotic stewardship programs. While they have been effective, as evidenced by the reduction in CDI incidence over time, the risk of CDI is still not entirely mitigated, and there is a stark need for new, enhanced preventative strategies [28].

The utility of probiotics in preventing CDI has been a recurrent and controversial topic of discussion in literature. There has been data to support both using and avoiding probiotics, though the current American College of Gastroenterology guidelines do not recommend probiotics for the prevention of CDI [29, 30]. Furthermore, a 2021 review article ultimately found no definitive evidence to suggest that probiotics are an effective means for preventing CDI [31]. Similarly, prophylactic antibiotics for CDI have been considered in several studies, however, evidence is limited mainly to recurrent CDI and does not support regular use, particularly in older, more immunocompromised patients, such as those who typically undergo CABG [31]. A novel area of research in CDI prevention is toxin-based vaccination, although multiple attempts have failed to demonstrate a decreased incidence in CDI among high-risk individuals, despite safe tolerance of the vaccine [32]. There are no currently approved vaccines available for CDI prevention.

One potentially promising area of developing research is the utilization of anti-inflammatory or anti-rheumatic drugs as possible antimicrobial agents. One such agent is auranofin, a trialkylphosphine gold complex antirheumatic agent. Auranofin has shown strong antimicrobial activity against *Clostridioides difficile* in both *in vitro*, and *in vivo* animal studies [33]. There are no human studies examining this effect at the time of this study; however low-dose auranofin was shown to significantly protect against CDI in mouse models, with similar effects to vancomycin when used at a higher dose [34]. Other innovative strategies for CDI prevention include newer antibiotics, monoclonal antibodies, and fecal microbiota transplants. However, these are generally indicated for recurrent CDI episodes, rather than initial occurrence. In general, there are relatively few options for prevention of CDI apart from the current standards of practice in hand hygiene and antibiotic stewardship. Future research should continue to be directed toward innovative mechanisms of prevention.

### Limitations

The analysis is retrospective and due to the nature of large database studies, cannot explicitly account for unmeasured confounders. Although we adjusted for demographic, comorbidity, and hospital-level factors, the NRD does not capture key clinical variables such as perioperative antibiotic regimens, mechanical ventilation duration, illness severity scores, operative complexity, cardiopulmonary bypass time, or institutional infection control practices. These unmeasured factors are well-established risk determinants for both CDI and postoperative complications and may partially explain the observed associations. Because of this, we are also not able to assess specific perioperative antibiotic regimens to determine if certain protocols were more commonly associated with, or preceding, CDI. We are also unable to evaluate if other medications commonly associated with CDI were used prior to the development of the infection. Some literature suggests that prolonged ICU stay is another risk factor associated with CDI. However, because of the lack of explicit ICD-10 codes for ICU stay, we are not able to elucidate this effect and characterize it further.

Additionally, the NRD does not provide information on the timing of CDI diagnosis relative to surgery or other postoperative complications, nor does it distinguish between hospital-acquired and community-onset CDI. As a result, the temporal sequence between CDI and adverse outcomes cannot be established, limiting conclusions regarding directionality.

### Conclusions

The incidence of CDI among patients undergoing CABG has shown a significant decline over the 7-year study period from 2016 to 2022, reflecting potential improvements in infection control and perioperative practices. However, despite this encouraging trend, patients who do develop CDI remain at significantly elevated risk for adverse outcomes, including higher mortality, readmission rates, severe complications, and increased healthcare resource utilization. These findings highlight the need for continued vigilance and targeted strategies to identify and support this high-risk subgroup in order to prevent poor postoperative outcomes.

## Data Availability

The data that support the findings of this study are available upon reasonable request from the corresponding author.

## Abbreviations

AKI: acute kidney injury
CCI: Charlson Comorbidity Index
CDI: *Clostridioides difficile* infection
CABG: coronary artery bypass grafting
HCUP: Healthcare Cost and Utilization Project
ICD-10: International Classification of Diseases, 10th Revision
LOS: length of stay
NRD: Nationwide Readmissions Database
